# Frequency and geographic distribution of TERT promoter mutations in primary hepatocellular carcinoma

**DOI:** 10.1186/s13027-017-0138-5

**Published:** 2017-05-19

**Authors:** Francesca Pezzuto, Luigi Buonaguro, Franco M. Buonaguro, Maria Lina Tornesello

**Affiliations:** Molecular Biology and Viral Oncology Unit, Istituto Nazionale Tumori IRCCS “Fondazione G Pascale”, 80131 Napoli, Italy

**Keywords:** Telomerase, TERT promoter mutations, Hepatocellular carcinoma, Hepatitis B virus, Hepatitis C virus

## Abstract

Primary hepatocellular carcinoma (HCC) mainly develops in subjects chronically infected with hepatitis B (HBV) and C (HCV) viruses through a multistep process characterized by the accumulation of genetic alterations in the human genome. Nucleotide changes in coding regions (i.e. TP53, CTNNB1, ARID1A and ARID2) as well as in non-coding regions (i.e. TERT promoter) are considered cancer drivers for HCC development with variable frequencies in different geographic regions depending on the etiology and environmental factors. Recurrent hot spot mutations in TERT promoter (G > A at-124 bp; G > A at −146 bp), have shown to be common events in many tumor types including HCC and to up regulate the expression of telomerases. We performed a comprehensive review of the literature evaluating the differential distribution of TERT promoter mutations in 1939 primary HCC from four continents. Mutation rates were found higher in Europe (56.6%) and Africa (53.3%) than America (40%) and Asia (42.5%). In addition, HCV-related HCC were more frequently mutated (44.8% in US and 69.7% in Asia) than HBV-related HCC (21.4% in US and 45.5% in Africa). HCC cases associated to factors other than hepatitis viruses are also frequently mutated in TERT promoter (43.6%, 52.6% and 57.7% in USA, Asia and Europe, respectively). These results support a major role for telomere elongation in HCV-related and non-viral related hepatic carcinogenesis and suggest that TERT promoter mutations could represent a candidate biomarker for the early detection of liver cancer in subjects with HCV infection or with metabolic liver diseases.

## Background

Primary liver cancer is one of the commonest and deadliest malignancies in the world accounting for 782,000 new cases and 746,000 deaths in 2012 [1]. The highest incidence has been observed in men from Eastern and South-Eastern Asia (age standardized rates [ASR] 31.9 and 22.2 per 100,000, respectively) and in women from Eastern Asia and Western Africa (ASR 8.1 and 10.2 per 100,000, respectively). On the other hand, liver cancer incidence is intermediate in southern Europe and northern America (ASR 9.5 and 9.3/100,000 men, respectively), and low in western and northern Europe (ASR <7.5/100,000 men and <2.5/100,000 women) [2].

Hepatocellular carcinoma (HCC) and intrahepathic cholangiocarcinoma (ICC) are the most common histotypes of primary liver cancer accounting for about 80% and 15%, respectively, of all cases worldwide [3–5]. HCC and ICC mainly develops in patients with liver cirrhosis caused by chronic infection with hepatitis B (HBV) and hepatitis C (HCV) or caused by alcohol excess, as well as in patients with non-alcoholic fatty liver disease or other metabolic liver disorders [6]. HBV chronically infects more than 300 million people in the world, mainly in Asia and Africa, while HCV infects approximately 180 million people, mostly in Japan, Europe and United States [6]. Accordingly, HBV-related HCC are more frequent in Asia and Africa (above 50% of all cases), while HCV-related HCC are predominant in Europe and USA (35-50% of all cases) [2, 7, 8].

The complex multistep process of liver carcinogenesis includes inflammation, hepatic damage, cirrhosis, increased liver fibrosis and HCC [9–11]. The molecular mechanisms involved in the malignant transformation of hepatocytes are extremely complex and comprise numerous genetic and epigenetic alterations [12, 13]. Genome instability, mainly involving gains in chromosomes 1q, 5, 6p, 7, 8q, 17q and 20 and losses in chromosomes 1p, 4q, 6q, 8p, 13q, 16, 17p and 21, has been observed in more than 80% of HCC associated to chronic viral hepatitis [14–17].

Several lines of evidence suggest that the pattern of somatic mutations in liver cancer varies in different geographic regions very likely depending on environmental factors or host genetic diversity [18–21]. Indeed, tumor protein 53 (TP53) coding gene mutations in HCC have been observed to occur most commonly in sub-Saharan Africa and Southeast Asia, where the combination of dietary aflatoxin B1 (AFB1) exposure and hepatitis B infection promotes high rate of mutagenesis in the liver [22]. More recently, several new recurrent mutations affecting genes involved in cell cycle regulation and chromatin remodeling have been discovered by whole exome sequencing technology and found differentially distributed in different populations [23–26].

Moreover, the analysis by whole-genome sequencing allowed to discover a substantial fraction of recurrent somatic mutations in non-coding regions of human genome with important regulatory effects on the gene expression in cancer [27]. The most notable example has been the identification of hot spot activating mutations in the promoter region of telomerase reverse transcriptase (TERT) gene in about 85% of human tumors, including liver cancer [28–31]. The newly described mutations at nucleotides 124 (mostly G > A and rarely G > T) or 146 (G > A) before the ATG start site in TERT promoter region have been recognized as frequent and early alterations in the hepatic carcinogenesis [31, 32]. These mutations create a binding site for transcription factors ETS (E-twenty six) and ternary complex factor (TCF), causing TERT over expression and restoring the telomerase activity [33].

Moreover, the single nucleotide polymorphism rs2853669, located at −245 bp upstream of the ATG start codon in TERT promoter, has also shown to deregulate the expression levels of TERT mRNA [34].

We performed a systematic review of published studies to investigate the frequency of TERT promoter mutations in 1939 HCC with diverse etiologies. Moreover, we evaluated the mutational pattern of TERT promoter in tumors from different geographic areas to possibly correlate the type of nucleotide changes with specific environmental or genetic factors in different regions of the world.

### Telomerase and liver diseases

TERT gene encodes for the catalytic subunit of the telomerase reverse transcriptase which is an RNA-dependent DNA polymerase highly expressed in germ cells, in stem cells and in cancer cells [35, 36]. The telomerase synthesizes telomeres which are long stretches of 5’-TTAGGG-3’ DNA repeats ending in a single-strand 3’ G-rich sequence located at the extremities of human chromosomes. Telomeres protect chromosomes from degradation, end-to-end fusion and recombination and act as an internal clock by regulating the maximal number of cell replication and aging [37–43].

The pathogenesis of liver diseases is strongly dependent on telomeres length and telomerase expression [44]. Several studies have shown a relationship between cirrhosis and telomeres attrition suggesting that this event could be considered a marker of cirrhosis [45–47]. However, telomerase activity and telomere elongation is restored in up to 90% of HCC, compared to the 21% of adjacent non-tumor tissues [8, 48–50]. Moreover, long telomeres and increased telomerase levels have shown to be associated with aggressive HCC phenotype and with poor prognosis [51].

Telomerase is activated by different mechanisms during liver carcinogenesis. In HBV related HCC the telomerase reactivation is frequently caused by the insertion of the HBV DNA within or upstream the TERT gene [52–56]. Sung et al. identified integrated HBV DNA in 86.4% of liver cancers, by whole-genome deep sequencing, and found that genes recurrently affected by HBV integration were TERT (23.7%), myeloid/lymphoid or mixed-lineage leukemia 4 (MLL4) gene (11.8%) and cyclin E1 encoding gene (CCNE1) (5.2%) [57]. Totoki et al. performed a comprehensive transancestry liver cancer genome study on 506 HCC cases from Asia and USA and observed HBV integration in TERT locus in 22% of tumors [31]. Moreover, they observed that TERT promoter mutations were in general mutually exclusive with HBV genome integration in the TERT locus and with TERT focal amplification, suggesting that either event is sufficient to activate telomerases. In addition, Zhao et al. reported that HBV insertional sites are significantly enriched in the proximity of telomeres in HCC DNA but not in non-tumor cell genomes suggesting that the integrated virus in cancer tends to target chromosomal elements critical for the maintenance of chromosome stability [58]. Moreover, Yang et al. analyzed 2199 HBV integration sites and observed that affected genes included 23.1% of protein-coding genes and 24.7% of long noncoding RNAs (lncRNA) [59]. Interestingly, the most frequently lncRNA genes affected by HBV integration were related to telomere maintenance, protein modification processes, and chromosome localization [59].

In HCV-related HCC and non-viral related HCC the telomerase activation is due to TERT promoter mutations in 40% to 75% of HCC cases, however with a considerable variation in different cohorts, as detailed in the next section.

### TERT promoter mutations in different geographical regions

**Fig. 1:**
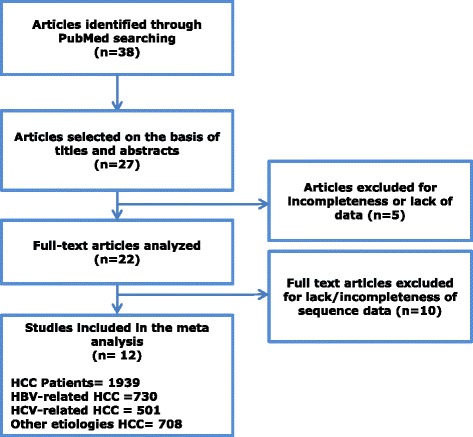
Flow diagram of selected articles and inclusion in the meta analysis

Published data on the analysis of TERT promoter mutations in liver cancer were searched in Medline using the terms (“hepatocellular” OR (“Liver” AND “Cancer”)) AND (“TERT” OR “telomerase”) AND (“Promoter”) AND (“mutation” OR “variation”), (Fig. 1). For the studies that involved more than one geographic location the data were divided into components for each continent. The search was updated on 31 January 2017.

The frequencies of TERT mutations in HCC have shown to vary by cancer etiology and geographic patient provenance (Table 1). Cevik et al. analyzed TERT promoter mutations in 15 HCC cases from Africa [60]. African patients comprised mainly HBV-positive subjects from Mozambique (*n* = 6), Transkei (*n* = 4), Lesotho (*n* = 2), Swaziland (*n* = 1) and South Africa (*n* = 2). The overall frequency of TERT promoter mutation among the HCC African cases was 53.3% and in the subgroup of HBV-related HCC was 45.5%. No other study has analyzed the TERT promoter mutation pattern in African HCC and more cases need to be analyzed to confirm such results.

**Table 1:** Distribution of TERT promoter mutations in HCC, associated to different etiologies, from divers geographic regions

Patients (*n =* 1939)		HBV+ patients (*n =* 730)	**HCV+ patients (** ***n*** = ***501*** **)**	Other etiol. (*n =* 708)	TERTp mut (*n =* 929) (%)	−124 hotspot (*n =* 869) (%)	−146 hotspot (*n =* 43) (%)	HBV+ mut (*n =* 227) (%)	HCV+ mut (*n =* 313) (%)	Other etiol. mut^a^ (*n =* 389) (%)	Article
AFRICA	Lesotho (*n = 2*)	2			1 (50)		1 (100)	1 (50)			Cevik et al., 2015 [60]
	Mozambique (*n = 6*)	5		1	4 (66.6)	2 (50)	2 (50)	3 (60)		1 (100)	Cevik et al., 2015 [60]
	South Africa (*n = 2*)	2			1 (50)	1 (100)		1 (50)			Cevik et al., 2015 [60]
	Swaziland (*n = 1*)	1									Cevik et al., 2015 [60]
	Transkei (*n = 4*)	1		3	2 (50)	2 (100)				2 (66.6)	Cevik et al., 2015 [60]
	**Total cases (** ***n*** = ***15*** **)**	**11**		**4**	**8 (53.3)**	**5 (62.5)**	**3 (37.5)**	**5 (45.5)**		**3 (75)**	
AMERICA	USA - African – Americans (*n = 12*)	2	7	3	8 (66.6)	8 (100)		1 (50)	5 (71.4)	2 (66.6)	Killela et al., 2013 [61]^b^
	USA - African – Americans (*n = 11*)	1	9	1	1 (9.1)	1 (100)			1 (11.1)		Totoki et al., 2012 [31]
	USA - Asian Ancestry (*n = 14*)	8	4	2	5 (35.7)	4 (80)	1 (20)		4 (100)	1 (50)	Totoki et al., 2012 [31]
	USA – Asian Ancestry (*n = 8*)	7		1	2 (25)	2 (100)		1 (14.3)		1 (100)	Killela et al., 2013 [61]^b^
	USA - European Ancestry (*n = 50*)	3	32	15	21 (42)	20 (95.2)	1 (4.8)	2 (66.6)	13 (40.6)	6 (40)	Totoki et al., 2012 [31]
	USA – European Ancestry (*n = 24*)	1	7	16	12 (50)	12 (100)			4 (57.1)	8 (50)	Killela et al., 2013 [61]^b^
	USA – Unknown Ancestry (*n = 17*)	5	2	10	5 (29.4)	4 (80)	1 (20)	2 (40)	1 (50)	2 (20)	Killela et al., 2013 [61]^b^
	USA – Unknown Ancestry (*n = 14*)	1	6	7	6 (42.8)	6 (100)			2 (33.3)	4 (57.1)	Totoki et al., 2012 [31]
	**Total cases (** ***n*** = ***150*** **)**	**28**	**67**	**55**	**60 (40)**	**57 (95)**	**3 (5)**	**6 (21.4)**	**30 (44.8)**	**24 (43.6)**	
ASIA	China (*n = 275*)	259		16	85 (30.9)	84 (98.8)	1 (1.2)	78 (30.1)		7 (43.7)	Yang et al., 2016 [74]
	China (*n = 35*)			35	11 (31.4)	9 (81.8)	2 (18.2)			11 (31.4)	Huang et al., 2015 [74]
	China (*n = 8*)	8									Cevik et al., 2015 [60]
	Japan (*n = 374*)	107^e^	139	128	224^b^ (59.8)	208 (92.8)	9 (4)	40 (37.4)	104 (74.8)	80 (62.5)	Totoki et al., 2014 [31]
	Japan (*n = 11*)			11	9 (81.8)	9 (100)				9 (81.8)	Ki et al., 2016 [77]
	Japan (*n = 11*)	1		10	4 (36.4)	3 (75)	1 (25)			4 (40)	Cevik et al., 2015 [60]
	South Korea (*n = 105*)	78	6	21	41 (39)	39 (95.1)	2 (4.9)	23 (29.4)	5 (83.3)	13 (61.9)	Lee et al., 2016 [76]
	Taiwan (*n = 195*)	12^e^	50	24	57 (29.2)	54 (94.7)	3 (5.3)	25 (20.6)	27 (54)	5 (20.8)	Chen et al., 2014 [73]
	**Total cases (** ***n*** = ***1014*** **)**	**574**	**195**	**245**	**431 (42.5)**	**406 (94.2)**	**18 (4.2)**	**166 (28.9)**	**136 (69.7)**	**129 (52.6)**	
EUROPE	France (*n = 305*)	67	68	170	179 (58.6)	168^c^ (93.8)	11 (6.1)	26 (38.8)	49 (72.1)	104 (61.1)	Nault et al., 2013 [69]
	France (*n = 193*)	24^e^	36	133	120^d^ (62.1)	106^c^ (88.3)	5 (4.2)	10 (41.6)	27 (75)	83 (62.4)	Schulze et al., 2015 [30]
	Germany (*n = 78*)			78	37 (47.4)	37 (100)				37 (47.4)	Quaas et al., 2014 [78]
	Germany (*n = 7*)	3		4	3 (42.8)	2 (66.6)	1 (33.3)	1 (33.3)		2 (50)	Cevik et al., 2015 [60]
	Italy (*n = 127*)	12^e^	110	5	64 (50.4)	62 (96.9)	2 (3.1)	5 (41.6)	59 (53.6)		Pezzuto et al., 2016 [32]
	Italy (*n = 41*)	10	20	11	21^d^ (51.2)	20 (95.2)		7 (70)	8 (40)	6 (54.5)	Schulze et al., 2015 [30]
	Spain (*n = 9*)	1	5	3	6 (66.6)	6 (100)		1 (100)	4 (80)	1 (33.3)	Schulze et al., 2015 [30]
	**Total cases (** ***n*** = ***760*** **)**	**117**	**239**	**404**	**430 (56.6)**	**401 (93.2)**	**19 (4.4)**	**50 (42.7)**	**147 (61.5)**	**233 (57.7)**	

^a^This group comprises HCC cases of various etiologies including alcohol intake, metabolic syndrome, NAFLD, NASH, hemochromatosis and cases with unknown etiology (Cevik et al., [60], Huang et al., [74], Killela et al., [61], Quaas et al., [78], Schulze et al., [30])

^b^This group comprises 4 cases of −57 T > G mutations, one case of −64 CG > TC substitution, one case of −69 C > A substitution and one patient showing contemporary −124 G > A mutation and of −116 G > T substitution (Totoki et al., [31])

^c^These groups comprise two (Nault et al., [69]) and one case (Schulze et al., [30]) of −124 G > T mutation, respectively

^d^These groups comprise 4 mutations −57 T > G, one substitution −53 A > G, one substitution g.1271232 A > G, one substitution 1293829 G > T and two cases of deletion (French cohort) and one case of g.1294963 G > A substitution (Italian cohort), respectively (Schulze et al., [30])

^e^These groups comprise twelve cases (Totoki et al., [31]), twenty cases (Chen et al., [73]) four cases (Schulze et al., [30]) and two cases (Pezzuto et al., [32]) of HBV+/HCV+ patients, respectively

Two studies evaluated TERT promoter mutations among 150 HCC cases from the United States and the overall mutation rate was 40% [31, 61]. The HCV-related HCC and non viral related cases, mainly associated to alcohol and metabolic syndrome, were more mutated (44.7% and 43.6%, respectively) compared to HBV-related cases (21.4%), Table 1. Both USA cohorts comprised patients with European ancestry (*n* = 74), Asian ancestry (*n* = 22) and African-American ancestry (*n* = 23). Comparable frequencies of TERT promoter mutations were observed between European (43.6%) and African (37.5%) HCV-related HCC. Larger studies are warranted in the USA to analyze the TERT variation frequencies in HBV-related and no-virus related HCC and to determine whether the genetic background has a role in the accumulation of TERT mutations in HCC in this multiethnic population.

In Asia, a total of 1014 HCC have been analyzed for TERT promoter nucleotide changes comprising 396 cases from Japan, 318 from China, 195 from Taiwan and 105 from South Korea. The overall mutation frequencies in TERT promoter were 28.9% in HBV-positive, 69.7% in HCV-related and 52.6% in non viral related HCC (Table 1). However, there were significant differences between mutation rates observed in HCV-related and no virus related HCC in Japan (74.8% and 62.4%, respectively) and South Korea (83.3% and 61.9%) versus Taiwan (54% and 20.8%). Similarly, variable rates of TERT mutations were observed among HBV positive HCC with high frequency in Japan (37.4%), intermediate in China (30.1%) and South Korea (29.4%) and low in Taiwan (20.6%).

In Europe, among the 760 HCC analyzed in five studies a total of 430 (56.6%) cases were found mutated in TERT promoter. The proportion of hot spot mutations in HCV, no virus and HBV related HCC was 61.5%, 57.7% and 42.7%, respectively. The highest mutation rate was observed in HCV-positive (73.1%) and other etiology HCC (61.7%), mainly related to alcohol, in France. In Italy, lower rates of TERT promoter mutations were observed in HCV-positive HCC, ranging from 40% to 53.6%, and in HBV-positive HCC, ranging from 70% to 41.6%, from northern and southern Italy patients, respectively.

In all studies the activating mutation at nucleotide −124 G > A was more frequent than the mutation at position −146 G > A (93.4% versus 4.6%, respectively).

### TERT promoter mutation and rs2853669 polymorphism

Several studies have reported that the single nucleotide polymorphism (SNP) rs2853669 allele G, located at nucleotide −245 from the TERT ATG start site, down regulates the expression of TERT gene caused by hot spot promoter mutations in several types of cancer including bladder, gliomas, and renal cell cancer [62, 63]. In the general population the rs2853669 allele G is less frequent than allele A, except for the south Asia population where it has been observed the reverse [64] (www.ncbi.nlm.nih.gov/projects/SNP/snp_ref.cgi?rs=2853669).

Only two studies evaluated the rs2853669 polymorphism and TERT promoter mutations in liver cancer. The study by Pezzuto et al., analyzed the allele frequency of TERT SNP rs2853669 in HCC from Southern Italy patients and showed allele frequencies of 51% A and 48.9% G among the TERT promoter mutated HCC and 57.6% A and 42.4% G among non-mutated cancer cases [32]. Although G allele appeared more frequent among TERT mutated cases, such difference did not reach statistical significance. Moreover, the Log-rank survival analysis showed no correlation between the presence of TERT promoter mutations, alone or in combination with rs2853669 GG and GA genotypes, and poor prognosis (*p* = 0.368) [32].

Ko et al. analyzed the impact of rs2853669 polymorphism in a cohort of south Korean HCC patients and observed no effect on the overall and recurrence-free survival. However, the combination of rs2853669 G allele and mutation in the TERT promoter was associated with poor survival [65]. Moreover, they showed that the rs2853669 nucleotide G causes increased binding of the transcription factor ETS2 to the TERT promoter and lower activity of the transcription inhibitor E2F1. This condition favors TERT promoter methylation and increased expression of telomerases [65]. Methylation of TERT promoter has been observed in several tumors and transformed cell lines and has been reported to correlate with TERT over expression and poor survival [66, 67].

## Discussion

Telomerase activity has been found strongly up regulated in many human cancers including HCC, highlighting its pivotal role in the neoplastic process [28, 48, 49, 68]. TERT promoter mutations have been recognized as the earliest and most frequent genetic alterations in liver cancer [25, 31, 69]. We have summarized the TERT promoter mutation distribution in HCC cases, associated to different etiologies, from various geographic regions.

In Africa, where HCC cases are mainly related to HBV infection and AFB1 dietary exposure, the frequency of TERT promoter mutations is around 53%. It is not known if there is synergistic effect between AFB1 and HBV on the accumulation of mutations in TERT as observed for the G to T variation at codon 249 in TP53 gene, specifically caused by HBV and AFB1 [70–72]. Interestingly, in USA where patients have no AFB1 exposure, the frequency of TERT promoter mutations among HBV-positive cases is 21.4% [31, 61]. In Asia, the overall rate is 42.5% with lower frequencies in China and Taiwan [31, 60, 73–77]. Higher frequencies of TERT promoter mutations, ranging from 42.8% to 66.6%, have been observed in Europe [30, 32, 60, 69, 78].

**Fig. 2:**
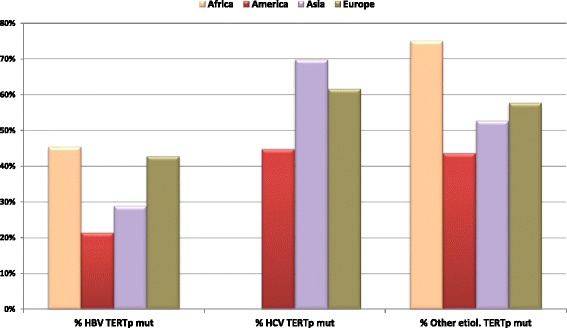
Frequency of TERT promoter mutations in all HCC from different geographic regions stratified by HBV (% HBV TERTp mut), as percentage of HBV+ HCC cases characterized by TERT promoter mutations, and HCV (% HCV TERTp mut), as percentage of HCV+ HCC cases characterized by TERT promoter mutations. Patients with both HBV and HCV infections have been included in the HBV group. HCC cases of various etiologies including alcohol intake, metabolic syndrome, NAFLD, NASH, hemochromatosis and cases with unknown etiology have been grouped in Other etiologies (% Other etiol. TERTp mut)

As shown in Fig. 2, HCV positive HCC have in general higher TERT promoter mutations rates than HBV positive tumors, in which TERT over expression is frequently caused by HBV integration [31, 32, 60, 73, 74]. HCC caused by non viral factors, such as alcohol consumption, metabolic syndrome, nonalcoholic fatty liver disease (NAFLD), nonalcoholic steatohepatitis (NASH), hemochromatosis, have a striking high frequency of mutation in TERT promoter. In fact, Ki et al. showed that in Japan 81.8% of NAFLD related HCC were mutated in TERT promoter [77]. In Europe, Nault et al. reported TERT promoter mutations in 68% of alcohol related HCC and in 63% of hemochromatosis related HCC cases [69].

Interestingly, TERT promoter mutations were more frequent in older patients [69, 73], and often associated with activating mutations in catenin beta 1 coding gene (CTNNB1) suggesting a cooperation between telomerase activity and β-catenin pathway [69].

## Conclusions

In conclusion, TERT promoter mutations are very frequent in HCC with different etiologies and are tumor specific given their constant absence in non-tumor tissues. There is a substantial heterogeneity in the mutation frequency in HCC from different geographic regions, probably due to environmental factors, such as AFB1, and lifestyle, such as habit of alcohol consumption. The high proportion of HCC mutated cases in different geographic regions and the earliness of occurrence of TERT mutations during hepatocarcinogenesis suggest the use of this reliable biomarker for early HCC diagnosis and as possible target for specific therapies.

## Abbreviations

AFB1: Aflatoxin B1
ARID1A: AT-rich interaction domain 1A coding gene
ARID2: AT-rich interaction domain 2 coding gene
CCNE1: Cyclin E1 coding gene
CTNNB1: catenin beta 1 coding gene
ETS: E-twentysix
HBV: Hepatitis B virus
HCC: Hepatocellular carcinoma
HCV: Hepatitis C virus
ICC: Intrahepathic cholangiocarcinoma
lncRNA: Long noncoding RNAs
MLL4: Myeloid/lymphoid or mixed-lineage leukemia 4
NAFLD: Nonalcoholic fatty liver disease
NASH: Nonalcoholic steatohepatitis
TCF: Ternary complex factor
TERT: Telomerase reverse transcriptase coding gene
TP53: Tumor Protein 53 coding gene
